# Complete Genome Sequence of the Deep-Sea Bacterium *Psychromonas* Strain CNPT3

**DOI:** 10.1128/genomeA.00304-13

**Published:** 2013-05-30

**Authors:** F. M. Lauro, T. K. Stratton, R. A. Chastain, S. Ferriera, J. Johnson, S. M. D. Goldberg, A. A. Yayanos, D. H. Bartlett

**Affiliations:** School of Biotechnology and Biomolecular Sciences, the University of New South Wales, Sydney, New South Wales, Australiaa; Marine Biology Research Division, Scripps Institution of Oceanography, University of California, San Diego, La Jolla, California, USAb; J. Craig Venter Institute, Rockville, Maryland, USAc

## Abstract

Members of the genus *Psychromonas* are commonly found in polar and deep-sea environments. Here we present the genome of *Psychromonas* strain CNPT3. Historically, it was the first bacterium shown to piezoregulate the composition of its membrane lipids and to have a higher growth rate at 57 megapascals (MPa) than at 0.1 MPa.

## GENOME ANNOUNCEMENT

*Psychromonas* strain CNPT3 was isolated from a decaying amphipod collected at a depth of 5,700 m in the central North Pacific Ocean (1) using a high-pressure enrichment and isolation technique.

The genus *Psychromonas* includes piezophilic (high-pressure adapted), halophilic (high-salt adapted), and psychrophilic (low-temperature adapted) species that are widely distributed in aquatic environments and are an important component of polar and deep-sea microbiota (2–9).

CNPT3 was initially assigned to the genus *Vibrio* based on physiological and morphological traits (10). After the description of the genus *Psychromonas* (11) and the advances of molecular taxonomy, it was possible to reassign CNPT3 to the order *Alteromonadales* (12) and the genus *Psychromonas* (13).

Genomic DNA was purified from 1.8 liters of stationary-phase culture. The cells were harvested, washed once in phosphate-buffered saline (PBS), and resuspended in 2.4 ml of nuclei lysis buffer (Promega). The extraction was completed following the protocol of the Wizard Genomic DNA kit (Promega). The DNA was further purified by resuspending in 1.2 ml of Tris-EDTA (TE) buffer, extracting once with 1.2 ml of phenol-chloroform (pH 8.0) and once with 1.2 ml of chloroform and then precipitating by centrifugation (16,000 × *g*; 10 min; 4°C) with 120 µl of sodium acetate (3M; pH 4.8) and 4 ml of ethanol (100%). A small aliquot was used for quantification and quality control.

An initial draft sequence was obtained by generating 2 Sanger libraries (a 3- to 4-kb plasmid and a 36- to 40-kb fosmid library) and one 454 library. Quality control, template production, sequencing, and hybrid assembly have been previously described (14).

The initial draft assembly of CNPT3 encompassed 2,945,265 bp in length and 175 contigs. Any genomic region not covered by at least two independent fosmid clones was manually sequenced with long-range and short-range PCRs. Contigs with unknown positions in scaffolds were linked using combinatorial PCR. With the addition of 107,145 bp, the finished genome is 3,052,410 bp in a single circular chromosome, with a 38.6% GC and 10 rRNA operons. Of its 2,580 protein-coding genes, 77.25% have a predicted function while 22.75% are hypothetical genes. Open reading frame prediction and automatic annotation were obtained through the NCBI PGAAP (http://www.ncbi.nlm.nih.gov/genomes/static/Pipeline.html).

The genome contains complete pathways for heterotrophic lifestyle such as the complete glycolytic and tricarboxylic acid (TCA) pathways. When the clusters of orthologous groups (COG) composition was compared to that of the sister species *Psychromonas ingrahamii* (15), it was found to be statistically enriched in genes for motility and chemotaxis (COG category N) and in particular methyl-accepting chemotaxis proteins (COG0840). Few phage proteins were found (two phage integrases and one prophage regulatory homolog) and no conjugation proteins, suggesting few opportunities for horizontal gene transfer. The genome also encoded a type B fatty acid synthase (FAS)-polyketide synthase (PKS) system (16) for the synthesis of polyunsaturated fatty acids such as eicosapentaenoic acid (EPA) (20:5, n-3) and docosahexaenoic acid (DHA) (22:6, n-3). *Psychromonas* CNPT3 has been shown to produce EPA and traces of DHA (17), and the resulting increase in membrane unsaturation might play a role in low-temperature and high-pressure environments (18). There was no evidence of the presence of a light-activated photolyase, which has been used as a hallmark for autochthony in the deep-sea bacteria (17).

### Nucleotide sequence accession number.

The whole-genome shotgun project was deposited in NCBI under the accession number CP004404.
